# PVB/PEG-Based Feedstocks for Injection Molding of Alumina Microreactor Components

**DOI:** 10.3390/ma12081219

**Published:** 2019-04-14

**Authors:** Anna Julia Medesi, Dorit Nötzel, Thomas Hanemann

**Affiliations:** 1 Karlsruhe Institute of Technology–KIT, Institute for Applied Materials, Hermann-von-Helmholtz-Platz 1, 76344 Eggenstein-Leopoldshafen, Germany; 2 Department of Microsystems Engineering-IMTEK, University of Freiburg, Georges-Koehler-Allee 102, 79110 Freiburg, Germany; dorit.noetzel@kit.edu (D.N.); thomas.hanemann@kit.edu (T.H.)

**Keywords:** ceramic injection molding, alumina feedstock, PVB-/PEG-based binder system

## Abstract

The ceramic injection molding (CIM) process is a cost-effective powder-based near net shape manufacturing process for large-scale production of complex-shaped ceramic functional components. This paper presents the rheological analysis of environmentally friendly CIM feedstock formulations based on the binder components polyvinyl butyral (PVB) and polyethylene gycol (PEG). The prepared PVB/PEG-based alumina molding compounds were investigated with respect to their PVB:PEG ratios as well as to their powder filling degrees in the range between 50 and 64 vol.%. Corresponding viscosities and shear stresses were determined for increasing shear rates to show the effects of increased PEG content and solid loadings on them. Two single reactor components were injection molded and subsequently joined in their green state for fabrication of an alumina microreactor. The intended purpose of the alumina microreactors is their potential application as wear-resistant and hydrothermal stable multifunctional devices (µ-mixer, µ-reactor, µ-analyzer) for continuous hydrothermal synthesis (CHTS) of metal oxide nanoparticles in supercritical water (sc-H_2_O) as the reaction medium.

## 1. Introduction

Powder injection molding (PIM) technology is a popular batch fabrication process for pure polymers or polymeric feedstocks with ceramic or metal particle load [1]. It enables cost-effective mass production of microstructured and complex-shaped components for high performance applications [2]. In recent decades, PIM also achieved a significant position as a manufacturing technique for high value-add products with high dimensional accuracy [3]. Using additive manufacturing technologies like PolyJet 3D printing technology for rapid tooling of polymer injection mold inserts, the PIM process also becomes attractive for design studies and rapid prototyping.

Ceramic injection molding (CIM) processes are generally divided into four steps: (1) compounding; (2) injection molding; (3) debinding; and (4) sintering [4,5]. Defects in the final parts created during injection molding cannot be eliminated during step three and four [5]. Thus, the quality and stability as well as the properties of final parts depend on the choice of a suited binder system [6]. Usually high-pressure is applied during PIM processes and the binder system serves a very important role [7]. During the injection procedure, the molding compound undergoes extremely high shear rates of up to 10^6^ s^−1^ which may lead to powder binder segregation or non-homogeneous binder extraction during the debinding step [1]. Hence, a homogeneous distribution of processed particles is crucial in order to avoid anisotropic shrinkage of the ceramic part resulting in cracks, porosity or distortions in the final part [1,8].

The rheological performance is determined through the interactions of the multi-component system [7]. Therefore, understanding the feedstocks flow behavior which shows complicated sensitivity to variations in shear rate is a key factor for assessment of the processing conditions [5]. A high solid concentration is advantageous for a successful sintering process. For complete cavity filling, a pseudoplastic flow and low viscosities at the processing temperature that relieves processability is required [5,9]. The objective is to provide polymer-powder mixtures with viscosities below 1000 Pa·s at the processing shear rate, pseudoplastic flow behavior and a high concentration of solids [5,9].

Standard molding compounds are usually based on long-chain olefines, mostly LDPE (low-density polyethylene) and short-chain waxes [6,7,10,11,12]. These two organic additives forming the binder system have the following functions. The high molecular polymer, also called the backbone polymer, carries the ceramic particles during the shaping process and gives the injection molded green ceramic part its dimensional stability and mechanical strength during demolding and debinding [6]. The low molecular organic additive acts as a plasticizer [7] between the long polymer chains of the backbone polymer to lower the feedstocks’ dynamic viscosity and provide the flowability required for complete cavity filling during injection of the feedstock into the mold [6]. Many types of binder compositions have been developed based on this environmentally friendly binder to injection molding for a variety of powders [13,14,15,16]. However, most of them require the use of organic solvents during the solvent debinding stage prior to thermal decombustion of the PE [4,16]. Paraffin wax is generally removed by dissolving the wax using hexane or heptane [4,17,18]. With regard to “Green Process Engineering”, which requires the integration of new environmentally friendly chemical routes and technical innovation to achieve green process development [19], alternative binder systems based on polyethylene glycols (PEG) C_2n_H_4n+2_O_n+1_ have been investigated in order to replace organic solvents like hexane and heptane. The development of an environmentally friendly feedstock binder system must consider the substitution of any organic solvent or even the solvent pre-debinding [4]. Low molecular PEG is soluble in water and can be easily debound via water-leaching [7].

PEG-based binder systems have been investigated in combination with backbone polymers like polymethylmethacrylate (PMMA), carnauba wax (CW) or polyvinyl butyral (PVB). Bleyan et al. devoted their efforts to understanding the molecular interactions between PEG and CW and their applicability as environmentally benign components of CIM binder systems [7]. Investigated reactive binder mixtures based on PMMA/MMA and small amounts of PEG 1500 (2 wt.%) for composite reaction molding exhibit an excellent feedstock viscosity measured at a shear rate of 10^3^ s^−1^ at a processing temperature of 220 °C of about 100 Pa·s. However, the combination of water-assisted pre-debinding and subsequent thermal debinding results in lower densities (98% of the theoretical density) of the sintered bodies than a pure thermal debinding (100% of the theoretical density) [4]. Tülümen et al. investigated a commercially available LDPE/PW-based standard binder formulation and an alternative PVB/PEG-based binder formulation based on the work of Weber et al. [20] in combination with a very fine alumina powder to compare the feedstocks’ final mixing torques and viscosities. For a solid content of 50 vol.%, the viscosities of the PVB/PEG-based molding compound (30–100 Pa·s) had half the values of the LDPE/PW-based feedstock (80–200 Pa·s) at shear rates between 10^2^ and 10^3^ s^−1^ [12].

Weber et al. published the rheological properties of PVB/PEG-based molding compounds for various filler loadings of submicrometer zirconia [6]. Surawatthana et al. used the PVB/PEG-based binder system to prepare porous alumina ceramics [16]. The ceramic feedstock contained PEG as the major fraction. Nevertheless, about 90% of the PEG could be removed by water leaching (45–60 °C, 4 h) and the PVB matrix (minor fraction) was still stable enough to maintain rigidity for the subsequent thermal treatment [16]. In 2018, PEG/PVB-based compounds were even used for robocasting of alumina parts [21]. Chuankrerkkul et al. successfully fabricated dental ceramic brackets by PIM from alumina using the partially water-soluble PEG/PVB binder system in a ratio of 80:20 (wt.%) in combination with 52 vol.% powder loading and a mean powder particle size of 1 µm. Despite the high amount of PEG, the PEG removal was successfully carried out in water at 40 °C [2]. This high PEG content (at the expense of the backbone polymer) is obviously feasible for small parts like dental brackets. For larger parts, higher PVB content might be required to achieve dimensional stability of the injection molded green bodies during further processing. Molecular masses of the binder components have not been mentioned as well as detailed rheological analysis of the feedstocks’ behavior at the processing temperature.

The alumina parts injection molded in the course of this work were developed to be further processed to chemically inert, pressure- and hydrothermal resistant microreactors. Alumina has generally been known as one of the most widely used advanced ceramics due to its excellent properties such as high hardness, high strength as well as its wear resistance and bio-inertness [2] and it is still the most widely used PIM ceramic [22]. The earliest PIM applications that date back to the 1930s are alumina parts [23]. Products find their applications in areas where extreme conditions are applied [24]. The alumina microreactors presented in this article will find their application in continuous hydro-thermal synthesis (CHTS) of metal oxide nanoparticles. The CHTS is green materials synthesis and a promising alternative to conventional processes to form nanoparticles through mixing of a cold aqueous metal salt solution with hot compressed water [25]. Suited reactors for CHTS have to withstand the harsh conditions required to bring the hot water stream in its near- or supercritical state (pc = 22.1 MPa, Tc = 647.1 °C) [26]. The structure of the injection molded microreactor green bodies (one half of the microreactor) consists of two reaction channel inlets and five electrode channels on each side of the reaction channel. The electrode pairs along the reaction channel are used for an integrated sensor system based on impedance measurements, enabling an in-situ monitoring of the nanoparticle formations progress.

In this work, PVB/PEG-based alumina feedstocks were investigated rheologically in terms of their solid load and the PVB:PEG ratio. Injected parts usually contain between 35 and 50 vol.% organic binder [9]. Feedstocks with powder loadings >50 vol.% (highly filled) were prepared using two different feedstock formulations (F1 and F2). F1-feedstocks contain PVB as the major fraction (50 vol.% of the binder system) while F2-feedstocks contain PEG as the major fraction of the binder. F1 feedstocks were investigated for alumina loads up to 64 vol.%. F2-feedstocks were tested for 55 and 58 vol.% alumina loads. The effects of the powder filling degree and the PVB:PEG ratio on kneading torque, the viscosity and the emerging shear stresses of the multi-material compound at a processing temperature of 160 °C are presented in this paper.

## 2. Materials and Methods

**Figure 1:**
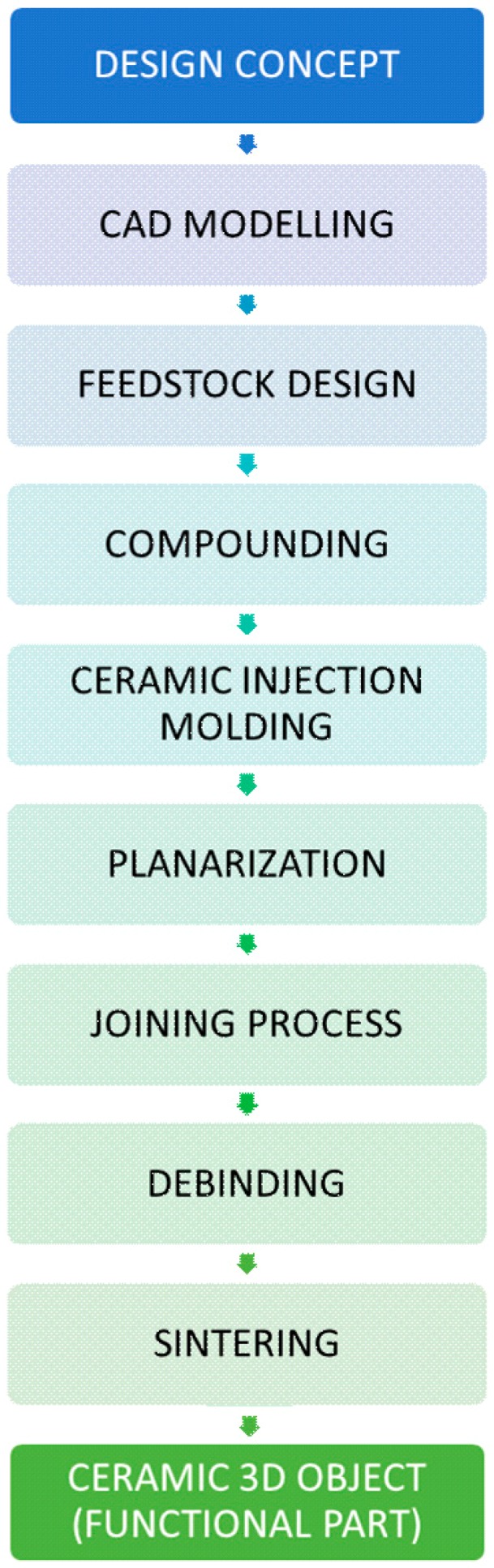
Process flow of the alumina µ-reactors production via ceramic injection molding (CIM) technology.

The ceramic microreactors for CHTS were fabricated according to the process chain depicted in Figure 1. A design concept for a flow-type microreactor with a simple T-mixer was developed by CAD modelling. Appropriate molding inserts were fabricated out of brass by micromilling. Several molding compounds with variations of the components’ quantities were prepared, investigated rheometrically, thermogravimetrically, dilatometrically and further processed via CIM. The temperature program for the subsequent thermal posttreatment (especially the heating rates for debinding) was tailored specifically to the components’ size.

### 2.1. Design Concept for the Microreactor

**Figure 2:**
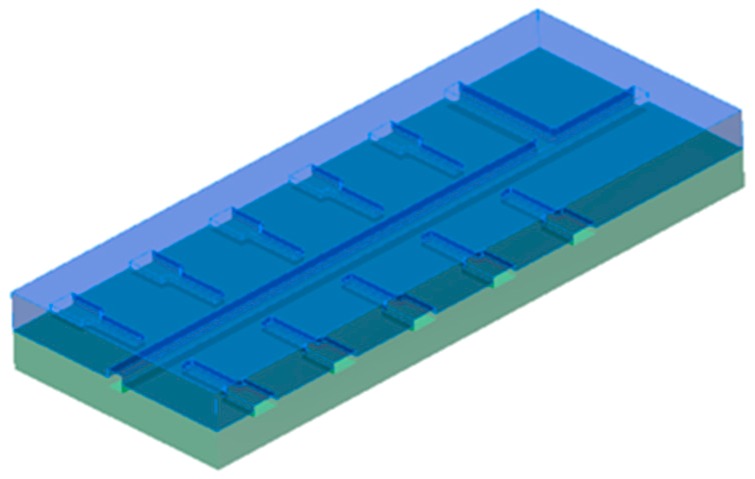
Design of a ceramic microreactor for continuous hydrothermal synthesis (CHTS) which is manufactured from two single parts (blue—top part, green—bottom part) using CIM and subsequent sinter-joining.

**Figure 3:**
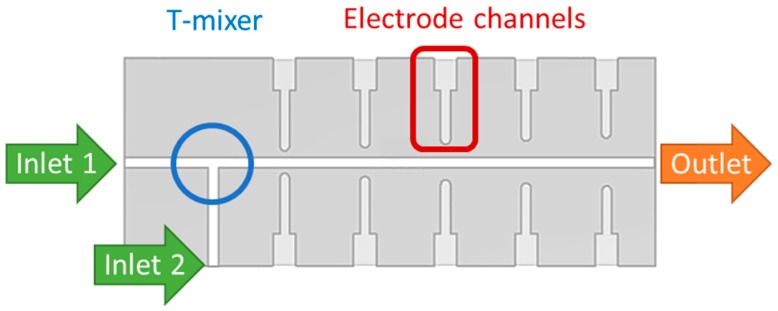
Electrodes along the reaction channel allow the integration of a sensor system that enables in-situ impedance spectroscopy measurements.

The microreactor is composed of two structured parts that have to be joined after the shaping process. Its structure consists of two inlet channels connected through a T-mixer to the reaction channel on which both sides five pairs of electrode channels are positioned at a maximum distance from each other. Figure 2 and Figure 3 depict the reactors’ design schematically and describe the functions of the channel structures.

### 2.2. Feedstock Design

The reactor material has to meet certain requirements to be suited for a CHTS. It must be chemically inert against aqueous metal salt solutions and also long-term resistant against supercritical water (sc-H_2_O). It requires a high pressure, corrosion and flow abrasion resistance, and besides, it should exhibit a low permittivity and it needs to be electrically non-conductive to avoid short circuits between the electrodes of the sensor system. Through the use of a high performance ceramic as a reactor material, wear protection can be achieved for the relevant conditions. Alumina is a high performance ceramic and is long-term hydrothermal resistant at high temperatures [27]. We used the fine-milled, sub-micron-sized alumina powder Martoxid^®^ MR70 (99.8% Al_2_O_3_, 0.08% SiO_2_, 0.06% MgO, Martinswerk^®^, Bergheim, Germany) with a mean particle size (d_50_) of 0.5 to 0.8 µm (d_90_ < 3 µm) and a specific surface (A_sp_) of 8.6 m^2^/g. The same alumina powder was used with a commercially available multi-component PE-based binder (Licomont EK 583-G, Clariant, Pratteln, Switzerland) in 2011 by Hausnerova et al. [5].

**Figure 4:**
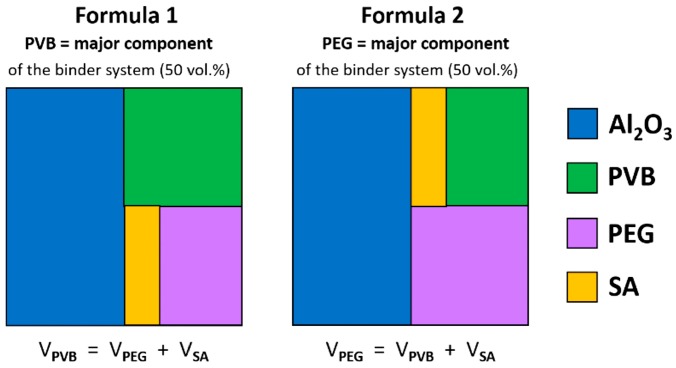
Component ratios (vol.%) of molding compounds with different binder component ratios for alumina feedstocks with 50 vol.% powder loading.

The alumina powder was mixed with several organic vehicles to get a well-suited molding compound for the CIM process. Polyvinyl butyral (PVB, Mowital B30 H, Kuraray Europe GmbH, Hattersheim am Main, Germany) was used as the structural polymer (M_n_ = 2.12 × 10^4^ g/mol). Polyethylene glycol (PEG 4000, Roth GmbH, Karlsruhe, Germany) was used as the basic polymer of the binder system. Stearic acid (Roth GmbH) was used as dispersant to achieve a steric stabilization of the alumina powder particles inside of the binder matrix. Its ratio was fixed to 4.4 mg/m^2^ of the powder surface. Figure 4 shows the two different PVB:PEG ratios that were investigated. The alumina content was varied from 50 vol.% up to 64 vol.% for Formula 1.

### 2.3. Compounding and Characterisation

All components were mixed at 125 °C in a measuring mixer (Plastograph, Brabender GmbH, Duisburg, Germany) for 1 h with 30 rpm. The molding compounds were characterized rheologically at a temperature of 160 °C which complies with the CIM processing temperature using a capillary rheometer (Rheograph 25, Göttfert GmbH, Buchen, Germany) with a nozzle diameter of 1 mm and a length-to-diameter (L/D) ratio of capillary of 30.

### 2.4. Ceramic Injection Molding

Reactor green bodies were fabricated from molding compounds with 55 and 58 vol.% Al_2_O_3_ using an injection molding machine (ELECTRA 50S, Ferromatik Milacron, Malterdingen, Germany) using micro-milled molding inserts made of brass. The parameters used for CIM are listed in Table 1. The injection speed is matched to the size and shape of the molded part and should generally be fast. However, to avoid contamination of the compound due to abrasion, the injection speed was set to 100 mm/s. To facilitate the demolding process, a silicone-free release agent (Z 265, Hasco, Lüdenscheid, Germany) was used to avoid the molded part sticking to the molding inserts’ surface. The holding pressure is required to control the dimensional stability [5].

### 2.5. Planarization

Before the two injection molded reactor parts were joined, the single green bodies were planarized thermally to cancel out any deformations or deflections that originate from the demolding step of the CIM process. For a reliable joint, the two parts must be completely plane-parallel to each other. Optically flat and uniformly even green bodies were obtained when they were heated up to 120 °C for 2 h between two alumina sintering plates (10 × 10 mm^2^).

### 2.6. Joining Process

The two reactor parts can be joined in their green state through dissolving the surfaces to be joined of both parts. Therefore, the green bodies’ structured surfaces were dissolved with isopropanol for a duration of 1 min to dissolve only a few microns of the surface. It was observed that longer residence times cause a loss of material at the joining seam since the compound mass that is partially dissolved at the surfaces becomes so soft that it is pushed easily out to the sides when the parts are stacked and pressed together. That is why dissolving the surfaces too deep leads to an unwelcome slot at the joining seam in the sintered body.

To achieve a properly sealed connection of the joining parts, it is necessary to perform the joining process with short residence times for the surface dissolving procedure and without much pressure. When the top and the bottom sections of the reactor are exactly on top of each other, they can be pressed together carefully. It is also possible to use ethanol as a solvent for the PVB-/PEG-compound. However, it must be taken into account that ethanol dissolves the green bodies’ surfaces even faster than isopropanol. Thus, it is recommended to join the parts immediately after wetting the surfaces with ethanol to hamper a loss of material along the joining seam.

### 2.7. Debinding Process

The thermal post-treatment process starts with a very slow combustion step where the organic components are burned out. To develop a suited temperature program for a smooth combustion of the PVB-/PEG-based binder without causing any defects in the ceramic microstructure, the thermal behavior of the molding compound that was used for manufacturing the microreactor parts (F2 with 55 vol.% powder loading) was investigated by thermal gravimetric analysis (STA 409, Netzsch, Selb, Germany). The stacked reactor parts were debound in a debinding furnace (HT6/28, Carbolite GmbH, Neuhausen, Germany) on top of an alumina sintering plate. Heating rates, holding temperatures as well as dwells were iteratively varied to develop a temperature program that also fits to the reactors’ size and factors in the thicknesses of 4 mm of each reactor part. Smaller components, like tribology disks, made of the same molding compound can be debound with much higher heating rates up to 5 K/min. since their aspect ratio is also higher than the one of the reactor assembly.

### 2.8. Sintering Process

Dilatometric measurements (Dil 402C, Netzsch, Germany) were applied to ceramic feedstocks with 55 vol.% as well as 64 vol.% powder load to determine the temperature at which the sintering process starts. The debound parts (brown bodies) were sintered in a sintering furnace (RHF 17/3, Carbolite, Germany) using a two-step-sintering process suggested by Chen and Wang (TSS-CW) to obtain ceramic bodies with a controlled microstructure [28]. The TSS technique is applied to create a fine-grained ceramic microstructure by suppressing the accelerated grain growth, which usually occurs in the final sintering stage, however it still allows densification to occur. The technique may thus enable high-density microstructure refinement and improve several properties of the materials. The applicability of the TSS technique as a means of suppressing the final stage grain growth was verified by Bodišová et al. for a sub-micrometer alumina powder (mean particle size = 200 nm) [29]. They yielded a high densification degree (ρ_rel_ = 98.8%) without any significant grain growth (mean grain size = 0.9 µm) with the TSS sintering plan: T1 at 1400–1450 °C and T2 at 1150 °C. Thereby, the first heating step T1 should be short at a relatively high-temperature in order to close porosity without significant grain growth. The second step T2 facilitates further densification with limited grain growth. The effect of heating rate was also examined.

Both density and grain size were lower if faster heating was applied. Kim and Kishi studied the effect of TSS on the resistance and the subcritical crack growth in alumina and found out that it led to decreased grain growth and a more homogenous microstructure which increased the fracture toughness of the grain boundary, resulting in strengthening the material resistance [30].

For sintering the reactor components during the course of this work, slightly higher temperatures for T1 and T2 were used than recommended by Bodišová et al. to address their larger size (8 × 26 × 66 mm^3^) in comparison to common specimens. T1 was set to 1500 °C and T2 to 1400 °C, while the holding times were varied. Before further processing of the sintered microreactor parts (applying electrodes for the sensor system to be integrated), it is necessary to check if the reactor channel is sealed. Possible residual leaks were capped through repeated painting (or using a dipping bath) of the sintered body into the commercial water-based impregnating agent for castings and RapidPrototyping models Nano-Seal 180W^®^ (JELN Imprägnierung GmbH, Schwalmtal, Germany). Nano-Seal 180W^®^ has a low viscosity and is thus able to penetrate into the pores due to capillary forces. It seals permanently elastic, so that the seal, which is additionally non-combustible, complies with the DIN EN 161 (tightness in gas valves).

## 3. Results

### 3.1. Effect of Powder Loading

**Figure 5:**
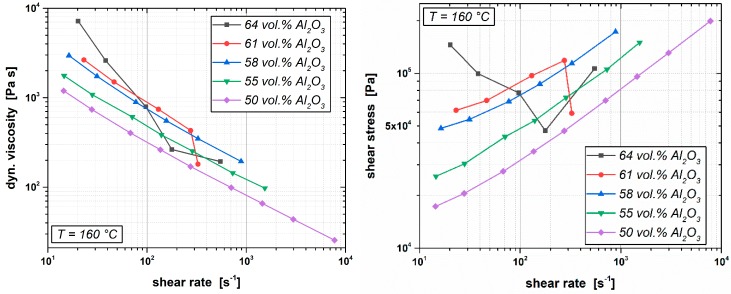
Rheological characterization of polyvinyl butyral- (PVB-)/polyethylene gycol (PEG)-based molding compounds with filler degrees of alumina powder between 50 vol.% and 64 vol.%.

The results of the rheological characterization of molding compounds according to formula F1 with different powder loadings is depicted in Figure 5. It shows the development of the compounds viscosity η and the arising shear stress τ while applying a certain shear rate $\dot{\gamma }$ at a typical CIM operating temperature of 160 °C. In general, typical shear rates used for CIM lie in the range between 10^2^ s^−1^ to 10^3^ s^−1^. The uniform correlation between shear rate and shear stress for feedstocks with up to 58 vol.% of Al_2_O_3_ powder shows that they are easily processable via CIM, even if shear rates higher than 10^3^ s^−1^ are used. If the filler degree is increased to 61 vol.%, the molding compound should be processed only with shear rates up to a maximum 300 s^−1^ since for higher shear rates, fluctuations (significant drop) in the compounds’ viscosity and the corresponding shear stress were detected. This effect is even more perceptible when the Al_2_O_3_ content is further increased up to 64 vol.%. Compounds with such high filling degrees do not show reliable rheological behavior anymore. Even at low shear rates (below 10^2^ s^−1^), the shear stresses do not progress gradually with a rising shear rate. Hence, for feedstocks referring to the binder system of formula F1, the maximum powder load lies below 60 vol.%, even though PVB/PEG-based molding compounds with higher filler degrees can easily be prepared and they are partially also readily processable via CIM if, for example, low shear rates are applied. During the course of this work, reactor green bodies from molding compounds with 62 vol.% of Al_2_O_3_ were also successfully injection molded. However, this high filler content is already near to the achievable limit for Formula F1. With respect to the size of the molded parts and to ensure a robust manufacturing process, we kept on injection molding compounds with lower filling degrees of only 55 and 58 vol.%.

**Figure 6:**
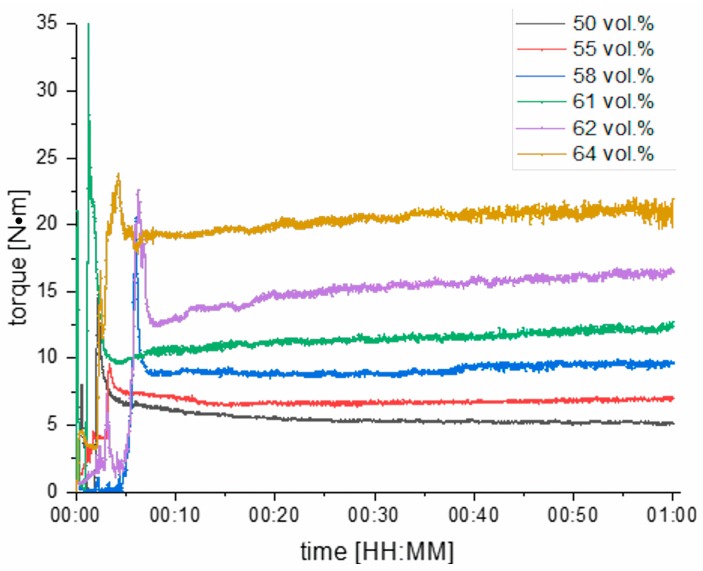
Mixing characteristics of PVB-/PEG-based molding compounds with different filler degrees of alumina powder.

Another important parameter to take into account in relation to the powder load of a molding compound is the torque that appears during the mixing procedure of the feedstock components. As shown in Figure 6, higher powder loads entail higher equilibrium torque. Additionally, the results of the mixing characteristics did not show that the mixing phase is significantly prolonged for higher filler degrees. A mixing phase of 30 min seems to be enough for all tested compounds. In Figure 6, the mixing characteristics of compounds with filler contents between 50 and 64 vol.% for a temperature of 125 °C are depicted and it appears that all compounds reach their equilibrium torque within the first 10 minutes of mixing. In contrast to the required mixing period, the mixing torque strongly depends on the filler content. Figure 6 shows that the equilibrium torque increases from 5 N·m for a feedstock with 50 vol.% powder content to a critical value of 20 N·m for a feedstock filled with 64 vol.% ceramic powder. This strong influence of powder content limits the maximum powder loading to less than 61 vol.% if an appropriate processability is to assure.

### 3.2. Effect of PEG Content on the Molding Compound Properties

**Figure 7:**
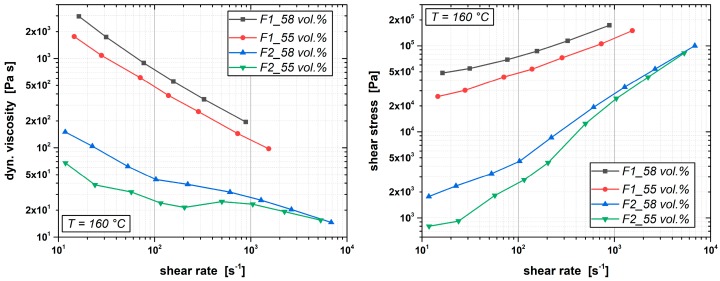
Rheological characterization of PVB-/PEG-based molding compounds with different quantities of the binder components (F1 and F2) for two different alumina powder filler degrees (55 vol.% and 58 vol.%).

The rheological behavior of feedstocks with different quantities of the binder components PVB and PEG was investigated for two different filler contents (55 and 58 vol.% Al_2_O_3_). The feedstocks´ compositions are given in detail in Table 2. The development of the viscosity as a function of the shear rate as the result of the rheological characterization is shown in Figure 7. It was observed that the viscosity of molding compounds at an operating temperature of 160 °C depends strongly on the ratio of the components of the binder system: The high-molecular back-bone polymer PVB (B30 H, kuraray) is needed to get robust green bodies which stay form stable during the debinding process too. Due to its high molecular weight ($\bar{M_{n}}=21.200$ g/mol, $\bar{M_{w}}=42.800$ g/mol, PDI = 2.022), it is the component that causes the pseudoplastic behavior of the molding compound while the low-molecular oligomer PEG 4000 that is also solid at room temperature, yet liquid at typical injection molding temperatures, significantly determines the viscosity at these increased temperatures. This correlation is also shown in Figure 7.

Feedstocks with increased PEG contents (formula F2) exhibit significant lower viscosities. In Table 2, the viscosity values are listed for the shear rates 10^2^ s^−1^ and 10^3^ s^−1^. It was found that, particularly at lower shear rates, the molding compounds according to Formula F2 that contain more PEG than PVB exhibit viscosity values that are significantly lower than the corresponding compound with the same powder loading and more PVB than PEG amount (F1). If the viscosities for a shear rate of 10^2^ s^−1^ are to compare, it can be seen that a higher PEG content (F2) reduces the viscosity of feedstocks with 55 vol.% powder loading from 499 Pa·s down to 31.6 Pa·s. For feedstocks with 58 vol.% powder load, the viscosity even drops from 798 Pa·s to only 57.7 Pa·s. This complies with a factor of about 15 ± 1 for both filling degrees. At higher shear rates, this ratio shrinks to about six times higher viscosity values for feedstocks with higher PVB contents. It is obvious that increasing the PEG content at the expense of the PVB content decreases the compounds’ viscosity markedly for equal filler contents. Hence, using higher amounts of PEG in the binder system makes the molding compound less viscous and enables theoretically higher powder loadings. Additionally, for low viscosities that facilitate form filling, complete form fillings can be achieved with less injection pressure.

Feedstocks with higher amount of the lower-molecular PEG concurrently exhibit significant lower shear stresses than feedstocks containing more of the backbone polymer PVB in the binder system. Lower shear stresses mean less abrasion during the injection procedure where the molding compound is pressed with 200–300 bar through the screw and the nozzle of the injection-molding machine into the cavity. Thus, this also means less contamination of abraded steel particles in the injection molded parts. Due to the macromolecules that are aligned during the shear procedure, molding compounds generally show non-Newtonian pseudoplastic behavior. So, the tested PVB-/PEG-based feedstocks also show this shear-thinning deviation from a linear Newtonian flow behavior. Viscosity and shear stress are not material constants depending only on temperature, concentration and pressure, however their values additionally depend on the shear rate that is applied. The Ostwald-de-Waele power-law describes the relation between shear stress τ and shear rate $\dot{\gamma }$ with the following equation of state:

(1) $$\tau =K\;\cdot {\dot{\gamma }}^{p}$$

The shear stress is defined as the applied force F per area A

(2) $$\tau =\frac{F}{A}$$

and the shear rate as the derivative of the deformation with respect to time:

(3) $$\gamma =\;\frac{dx}{dy}$$

(4) $$\dot{\gamma }=\;\frac{d\gamma }{dt}$$

The factor K, which is called the flow consistency index, and the flow behavior index p that presents the exponent in the Equation (1) were determined from the measured flow curves shown in Figure 7 by means of a non-linear fit. Table 3 contains the resulting values of K and p to compare the effect of the binder composition on the compounds’ flow behavior. The parameter p is a measure for the pseudo-plasitcity of a medium. The more the flow behavior index p differs from 1, the stronger the dependence of the shear stress on the applied shear rate is. It is marked that increasing the PEG content at the expense of the PVB content reduces not only the dynamic viscosity of the molding compound by a factor of about 6 to 16 (depending on the shear rate), however it also reduces the pseudoplastic behavior of the molding compound. At lower shear rates, this effect is more distinctive to notice: The higher the PEG content is, the more the flow behavior index p tends towards one (p → 1) which means that the flow behavior is less dependent on the shear rate and exhibits a more Newtonian flow behavior. In contrast, an increased PVB content leads to a more pseudoplastic flow behavior accompanied by a smaller flow behavior index (p < 1).

Hence, the PVB-/PEG-ratio of the binder system represents an applicable key to customize the flow behavior of a molding compound.

**Figure 8:**
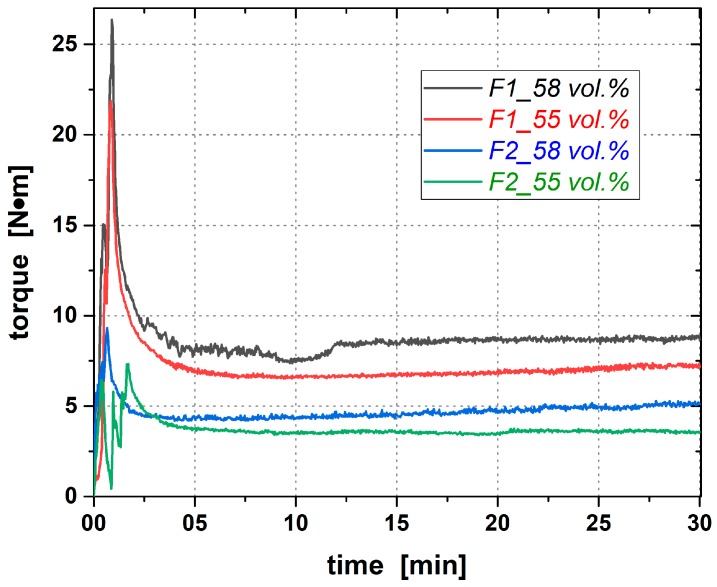
Mixing characteristics of PVB-/PEG-based molding compounds with different quantities of PVB- to PEG-ratio for filler degrees of 55 and 58 vol.% alumina powder.

This decrease of the molding compounds’ viscosity with increasing the amount of PEG also leads to lower required torques for mixing the components together, as it is shown in Figure 8. Regarding the mixing characteristics, it can be mentioned that all the four feedstocks can be mixed at 125 °C with lower end torques than 10 N·m (Table 4). However, the ratio of PVB to PEG has a strong effect on the mixing behavior, too.

### 3.3. Thermal Greenjoing versus Greenjoing with Dissolved Surfaces

The sintered parts show different qualities of joining seams depending on the joining method. When the two reactor parts were joined thermally by means of heating them up to 150 °C and joining them immediately by stacking them precisely and pressing them carefully together, as long as they were plasticized, the joining seams of the sintered bodies would not seal. The same can be said when the structured surfaces of both parts were dissolved for more than 1 min due to the slit that occurs at the joining seam on the account of a loss of material along the joining seam that arises when the upper and the lower parts are pressed together and the surfaces are dissolved too deeply. The best results could be achieved with short dissolving dwells of less than 1 min and by pressing the parts uniformly and carefully together with very low pressure.

### 3.4. Temperature Program for the Debinding Process

**Figure 9:**
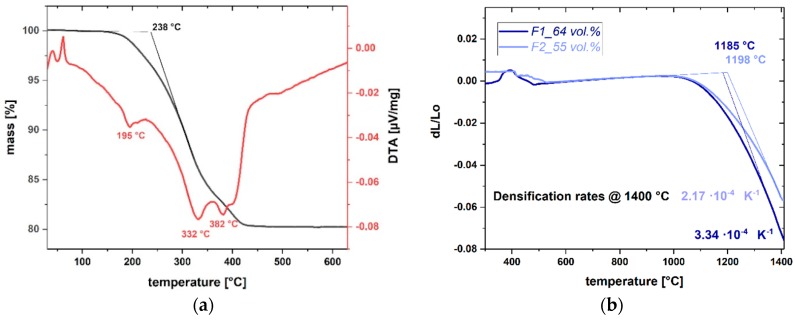
Thermogravimetric analysis of the feedstock according to formula II with an alumina powder load of 55 vol.% (**a**) and dilatometric measurements on alumina feedstocks containing 55 vol.% or 64 vol.% alumina powder (**b**).

The temperature program for the debinding process was customized to the thermal properties of the binder components. From previous thermogravimetric analysis on feedstocks with 50 vol.% alumina powder, shown in Figure 9, it is known that the thermal breakdown of the polymers starts already before 250 °C. The maximum loss of material of PVB-/PEG binder occurs around 400 °C. Its complete decomposition is given from around 600 °C. A suited temperature program also depends strongly on the components’ size. Programs for small parts can be driven with heating rates of 5 or 10 K∙min^−1^, however larger components with wall thicknesses of several millimeters need much lower heating rates to ensure a slow, steady combustion of the polymeric components and a reliable exhaust of the combustion gases through the fragile architecture of alumina particles. 

On the account of the thickness of the two reactor parts (2 × 4 mm^2^), heating of 0.1 to 0.2 K∙min^−1^ is required for their debinding process. Higher heating rates lead to visible defects in the microstructure of the brown bodies.

**Figure 10:**
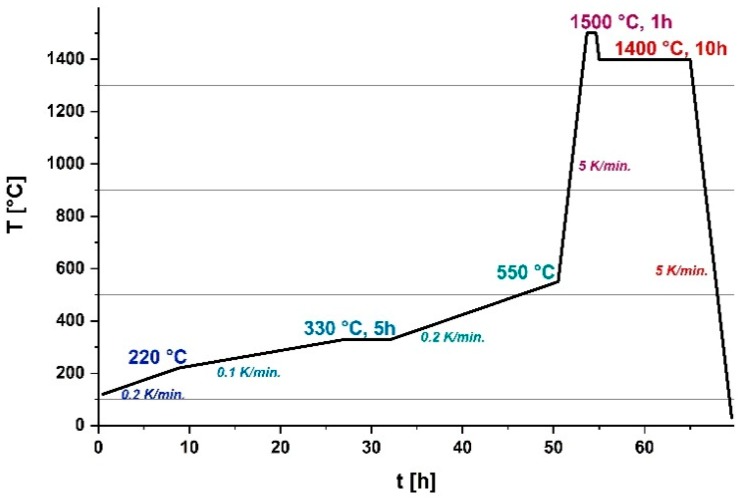
Temperature program for the debinding and sintering process of PVB-/PEG-based alumina green bodies in one go.

For this component size, it is crucial to minimize the heating rate down to 0.1 K∙min^−1^ before the pyrolysis starts to avoid bubble formation, microcracks and deflections. A dwell of 5 h at 330 °C is required to combust the major part of the binder components (>80 wt.%) and let the evolving gases uniformly out through the pores between the alumina particles. Figure 10 shows the total temperature program for the debinding and the sintering process of the µ-reactor green bodies in one go. Using this three-stage debinding profile, optically defect-free brown bodies were obtained. 

### 3.5. Temperature Program for the Sintering Process

During the sintering process, the component shrinks due to the closing of the pores between the alumina particles that occur during the debinding process. The unidirectional shrinkage of molding compounds (formula F1) with 55 and 64 vol.% alumina content was investigated by dilatometry. As Figure 9 shows, the densification of the Al_2_O_3_ particles starts around 1180 °C and reaches its maximum around 1210 °C for an alumina content of 55 vol.%. A higher filling degree of 64 vol.% facilitates the sintering of the particles and a slightly higher shrinkage rate is detected.

## 4. Conclusions

In this study, the rheological and thermal properties of CIM alumina feedstocks based on different ratios of the organic additives PVB and PEG were investigated to gain knowledge about their effect on the feedstocks’ viscosities and their pseudo-plasticity of highly filled molding compounds. Therefore, the filler content was also varied. As a result, it was shown that powder loads up to 64 vol.% can be realized even for the feedstocks containing more PVB than PEG, which makes them generally more viscous than feedstocks with higher PEG content. However, to ensure a robust manufacturing process including complete homogenization of the feedstocks components, complete cavity filling during injection as well as a good dimensional stability of the ceramic green bodies during demolding and debinding, filling degrees of 55 vol.% and 58 vol.% were selected for further investigations on the binder system and the CIM of alumina microreactors for CHTS.

The comparison of different formulations of PVB-/PEG-based CIM feedstocks shows that the low molecular binder component, PEG 4000, provides very low feedstocks viscosities at operating conditions of highly filled alumina compounds and, concurrently, sufficient mechanical strength of the molded green bodies at reduced temperatures. PEG 4000 can thus be used as the main share of the binder system without losing the required dimensional stability of the ceramic part. It was shown that the feedstocks viscosity at low share rates of 10^2^ s^−1^ and a processing temperature of 160 °C could be reduced from 499 Pa·s to 32 Pa·s (for the feedstock containing 55 vol.% alumina powder) and from 798 Pa·s down to 58 Pa·s (for a powder load of 58 vol.%) only by increasing the PEG content at the expense of the backbone polymer PVB. Furthermore, it was observed that adjusting the PVB-/PEG ratio is an applicable key to customize the flow behavior of ceramic feedstocks. 

Using the calculated flow consistency indices and flow behavior indices as a measure for the feedstocks pseudo-plasticity revealed that increasing the PEG content reduces the shear rate dependence significantly towards a more Newtonian flow behavior. In summary, it can be ascertained that feedstocks according to formula F1, containing more PVB than PEG, can be recommended for CIM of ceramic parts where the dimensional stability of the molded parts is challenging. However, massive part geometries with sufficient wall thicknesses can also be successfully manufactured using feedstocks according to formula F2, containing more PEG than PVB. These feedstocks have the advantage of providing very low feedstocks’ viscosities, which is desired to facilitate the cavity filling of challenging part geometries (high aspect ratios) with less injection pressure, which also means less abrasion and the potential for higher powder loadings.

## Figures and Tables

**Table 1:** Process parameters used for CIM of alumina compounds with filler degrees of 55 and 58 vol.%.

Injection Temperature (°C)	Injection Speed (mm/s)	Injection Pressure (bar)	Injection Time (s)	Holding Pressure (bar)	Holding Time (s)	Tool Temperature (°C)
160	100	286	0.57	210	2	30

**Table 2:** Viscosity at 160 °C (operating temperature) of alumina feedstocks with 55 or 58 vol.% powder loading based on binder systems containing 50 vol.% of the backbone polymer PVB (F1) or 50 vol.% of the lower molecular binder component PEG 4000 (F2).

Feedstock Formula		F1 (50 vol.% PVB/Binder System)	F2 (50 vol.% PEG/Binder System)	F1 (50 vol.% PVB/Binder System)	F2 (50 vol.% PEG/Binder System)
Al_2_O_3_	[vol.%]	55	55	58	58
Stearic acid	[vol.%]	8.3	8.3	8.8	8.8
PVB	[vol.%]	22.5	14.2	21.0	12.2
PEG	[vol.%]	14.2	22.5	12.2	21.0
Viscosity at 160 °C [Pa·s] at shear rate of 100 s^−1^		499	31.6	798	57.7
Viscosity at 160 °C [Pa·s] at shear rate of 1000 s^−1^		120	20.5	166	26.5

**Table 3:** Rheometrically determined indices of the Ostwald-de Waele relationship (Equation (1)) for molding compounds with different PVB-/PEG-ratios (Formula 1 and 2) and different powder loads.

Feedstock	Flow Consistency Index *K* [Pa·s^p^]		Flow Behavior Index *p* [dimensionsless]	
F1_58 vol.% Al_2_O_3_	15,367	(±1860)	0.353	(±0.020)
F1_55 vol.% Al_2_O_3_	7411	(±626)	0.407	(±0.013)
F2_58 vol.% Al_2_O_3_	256	(±22)	0.676	(±0.010)
F2_55 vol.% Al_2_O_3_	99	(±16)	0.785	(±0.019)

**Table 4:** End torque of Al_2_O_3_ feedstocks with different PVB:PEG ratios for a mixing temperature of 125 °C.

Powder Loading	55 vol.%		58 vol.%	
Feedstock formula	F1	F2	F1	F2
End torque/N·m at 125 °C (mixing temperature)	7.2	3.5	8.7	5.1
